# Universal non-circular cone beam CT orbits for metal artifact reduction imaging during image-guided procedures

**DOI:** 10.1038/s41598-024-77964-9

**Published:** 2024-11-01

**Authors:** Tess Reynolds, Yiqun Ma, Andrew Kanawati, Owen Dillon, Kenzie Baer, Grace Gang, Joseph Stayman

**Affiliations:** 1 University of Sydney, Sydney, Australia; 2 Johns Hopkins University, Baltimore, USA; 3 Westmead Hospital, Sydney, Australia; 4 OSSIS Corporation, Christchurch, New Zealand; 5 Univestiy of Pennsylvania, Philadelphia, USA

**Keywords:** Biomedical engineering, Imaging techniques

## Abstract

Innovation in image-guided procedures has been driven by advances in robotic Cone Beam Computed Tomography (CBCT) systems. A fundamental challenge for CBCT imaging is metal artifacts arising from surgical tools and implanted hardware. Here, we outline how two universal non-circular imaging orbits, optimized for metal artifact reduction, can be implemented in real-time on clinical robotic CBCT systems. Demonstrating potential clinical utility, the universal orbits were implemented during a pedicle screw cervical spine fixation and hip arthroplasty performed on a porcine and ovine cadaver respectively. In both procedures, the universal non-circular orbits noticeably reduced the metal artifacts surrounding the implanted orthopedic hardware, revealing anatomy and soft tissue obscured in current conventional CBCT imaging. This work represents a key step in clinically translating universal orbits, unlocking high quality in-room procedural verification to increase broader use of robotic CBCT systems and reduce the occurrence of secondary corrective surgeries.

## Introduction

Advances in robotic Cone Beam Computed Tomography (CBCT) imaging systems have facilitated their seamless integration into an ever-growing number of image guided interventions across a variety of disciplines^1–9^. However, one barrier that has persistently hampered an even wider adoption of interventional CBCT imaging is metal artifacts. That is, metal surgical tools or hardware attenuate incident X-rays, leading to photon starvation and beam hardening that ultimately results in artifacts in the reconstructed volumetric images. These metal artifacts can reduce the clinical utility of the images by obscuring anatomical features required to accurately guide, assess, or verify the performed intervention. As the challenge of metal artifacts in CBCT images is not new, there have been numerous approaches to address the issue.

Broadly speaking, approaches to reduce metal artifacts in CBCT images can be characterized as either algorithmic- or acquisition based. Starting with the algorithmic-based approaches, the focus has been on attempting to interpolate, or “in-paint”, in regions of incomplete data to reduce prominent streak artifacts in the reconstructed volumetric images^10–14^. The major limitations of current algorithmic-based approaches are that they are heavily dependent on the quality of the initially acquired information. That is, if the anatomy of interest is highly obscured during the acquisition, the accuracy of the interpolation algorithm will be low. As such, this inspired investigations into developing acquisition-based approaches that look to improve the quality of the acquired information via non-circular imaging orbits. The flexibility of modern robotic CBCT imaging systems provides the opportunity to realize non-circular imaging orbits more readily than ever before. Most non-circular imaging orbits for metal artifact reduction considered to date have been task-based, requiring some form of prior knowledge (e.g., shape/position of the metal tools/hardware and imaging task)^15–21^. While these highly optimized and personalized orbits have demonstrated the potential to provide high quality images, the practicality of integrating such orbits into clinical workflows remains uncertain. Specifically, prior knowledge of the patient, imaging task, and metal tools/hardware may not always be available.

**Fig. 1:**
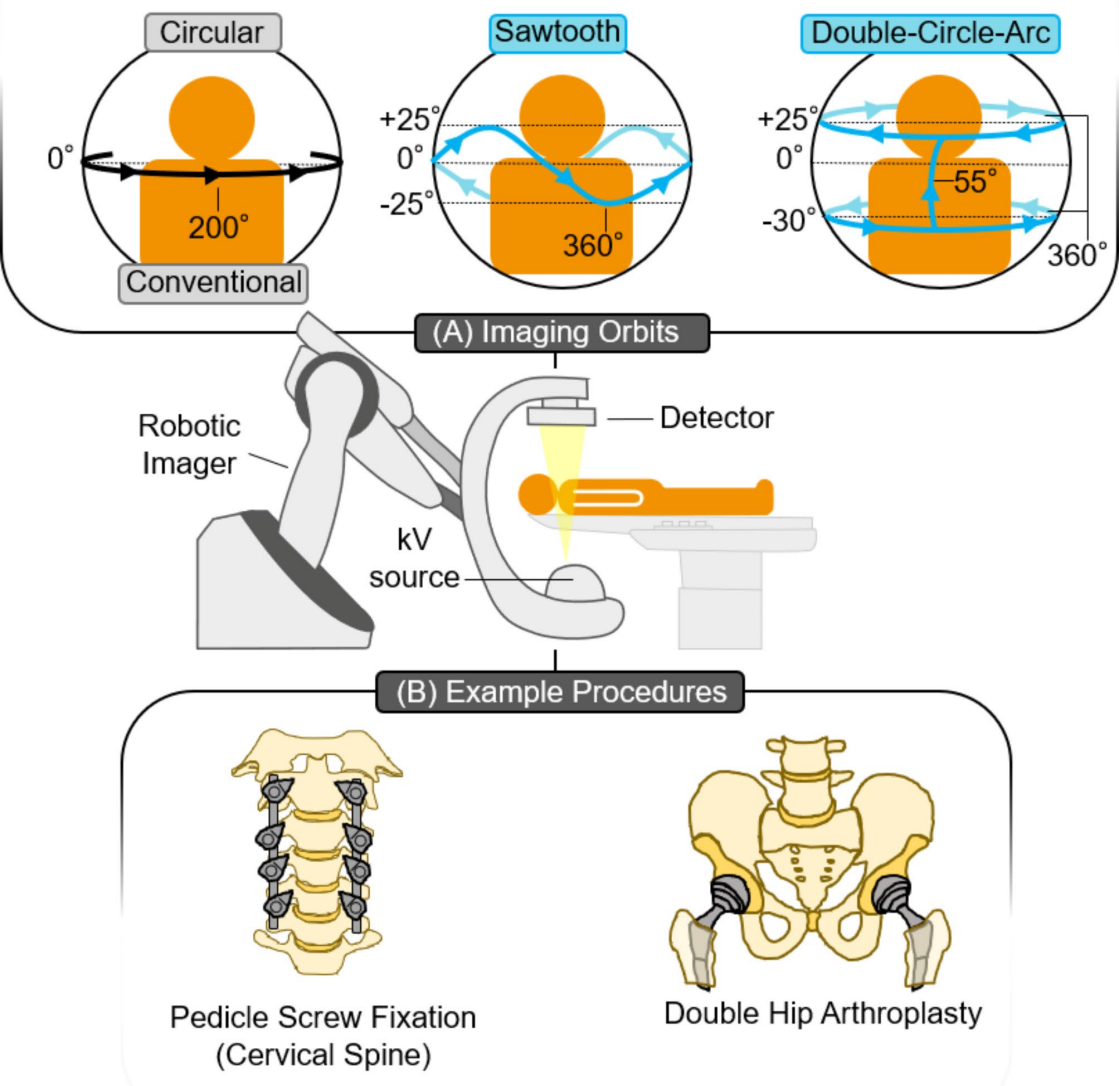
Overview of the CBCT imaging orbits and surgical procedures considered in this study. (**A**) Three imaging orbits were implemented on the robotic CBCT imager: circular (conventional), sawtooth and double-circle-arc. (**B**) Two surgical procedures, pedicle screw fixation in the cervical spine and a double hip arthroplasty, were performed on a porcine and ovine cadaver respectively.

Recently, universal non-circular orbits (referred to as universal orbits throughout) for metal artifact reduction have been proposed that don’t require any prior knowledge of the target anatomy, metal tools/hardware, or specific imaging task^22^. Specifically, Gang et al.^22^ sought source-detector trajectories that would be robust to metal artifacts, regardless of metal location. In short, this can be achieved with many different orbital designs which leverage redundancy in projection data to ensure the ‘missing data’ behind a metal object is always collected in an alternate opposing view. The orbit is ‘universal’ in the sense that a single orbital design can be robust for many interventional scenarios. Thus, universal orbits have the potential to fit seamlessly into existing clinical workflows, regardless of what anatomy, surgical tools/hardware, or procedures are being performed. Universal orbits for metal artifact reduction have previously been simulated^23^, implemented on a CBCT test bench set-up^24^, and implemented on clinical robotic CBCT imaging systems during phantom experiments^25,26^. Here, we look to implement two universal orbits in real-time on clinical imaging hardware and examine their potential clinical utility in two commonly performed, albeit distinct, orthopedic procedures. The orbits considered were a sawtooth and double-circle-arc. The procedures performed were a pedicle-screw fixation in the cervical spine of a porcine cadaver and a double hip arthroplasty in an ovine cadaver. An outline of the study is provided in Fig. 1.

## Results

### Image assessment – pedicle screw fixation

**Fig. 2:**
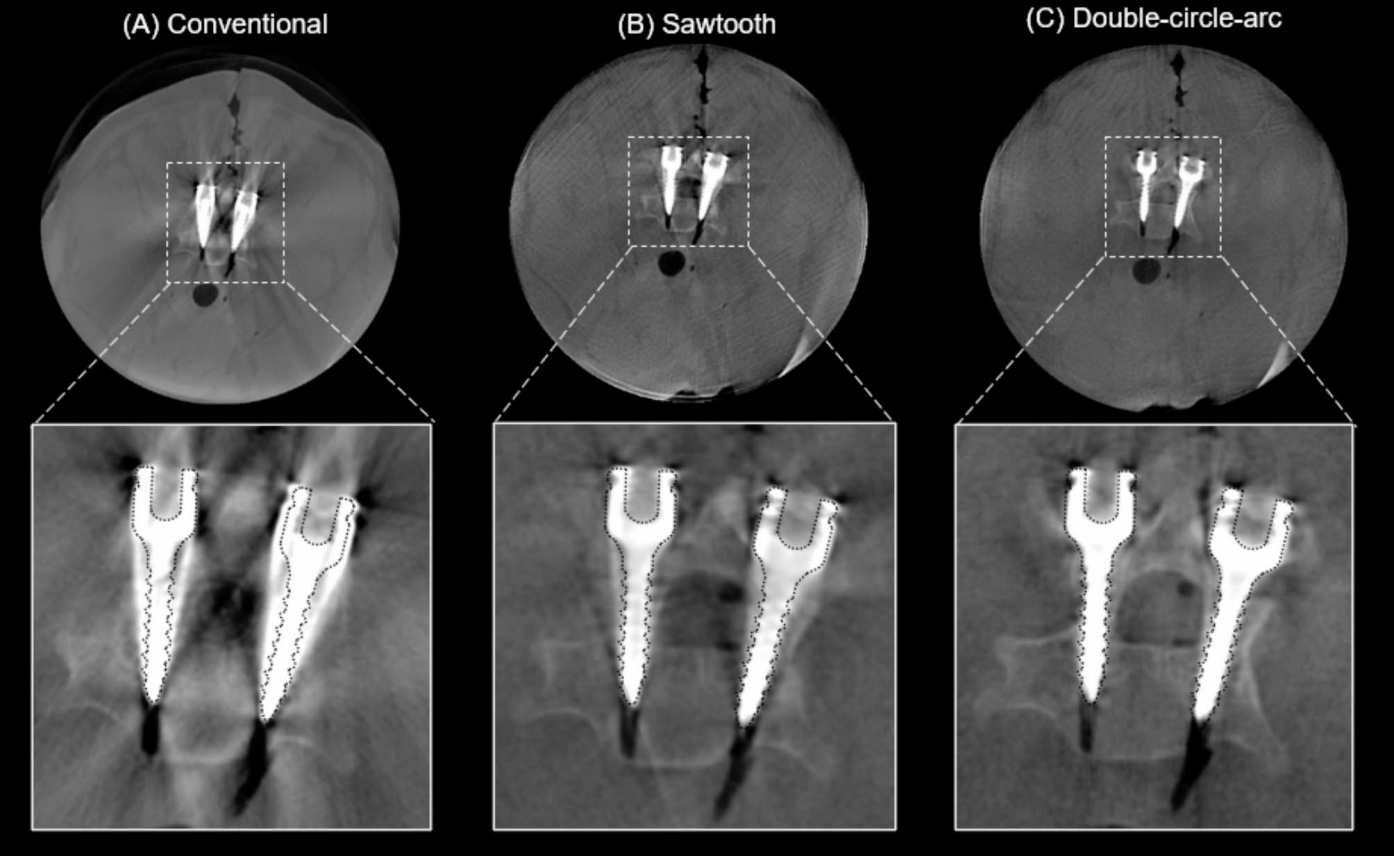
Post-operative reconstructed 3D images (axial view) of the C4 vertebra following pedicle screw fixation in the cervical spine of a porcine cadaver. (**A**) Conventional acquisition reconstructed with the in-built metal artifact reduction (MAR) algorithm, (**B**) the sawtooth acquisition and (**C**) the double-circle-arc acquisition. Grey value display: window width (W = 3392) and window level (L = 672). An outline of a model pedicle screw (dotted black line) has been overlayed onto the reconstructed images.

**Fig. 3:**
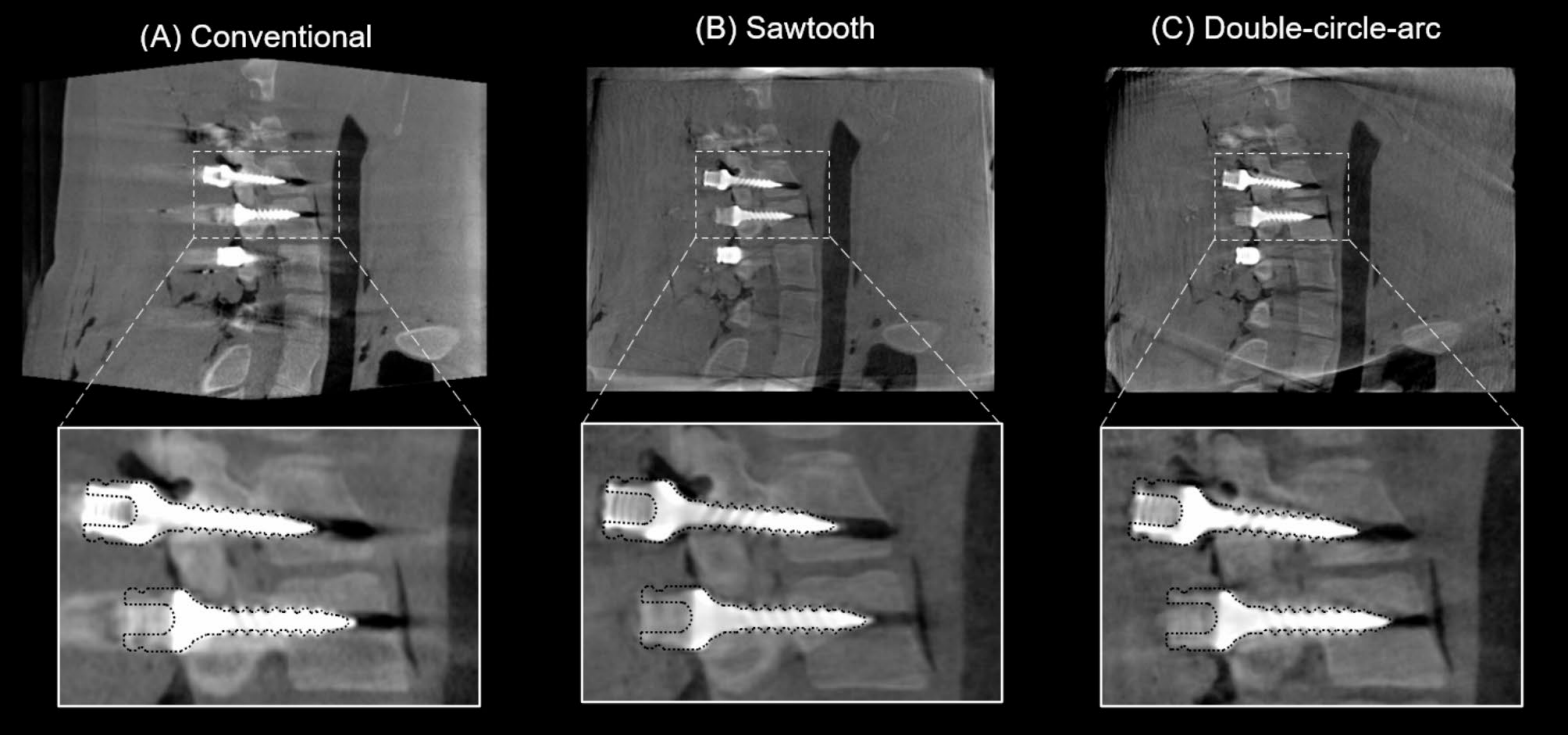
Post-operative reconstructed 3D images (sagittal view) following pedicle screw fixation in the cervical spine of a porcine cadaver. (**A**) Conventional acquisition reconstructed with the in-built metal artifact reduction (MAR) algorithm, (**B**) the sawtooth acquisition and (**C**) the double-circle-arc acquisition. Grey value display: window width (W = 3392) and window level (L = 672). An outline of a model pedicle screw (dotted black line) has been overlayed onto the reconstructed images.

Examples of the 3D reconstructed images from the pedicle screw fixation procedure in the porcine cadaver from the conventional, sawtooth, and double-circle-arc acquisitions are provided in Figs. 2 and 3 showing the axial and sagittal views, respectively. Considering the axial view shown in Fig. 2 first, there are significant artifacts arising from the pedicle screws in the conventional acquisition that are observably reduced with the sawtooth and double-circle-arc acquisitions, especially close to and surrounding the vertebra. In Fig. 3 showing the sagittal view, again there is observable reduction in the metal artifacts arising from the pedicle screws with both the sawtooth and double-circle-arc acquisitions compared with the circular conventional acquisition.

The observed reduction in metal artifacts was confirmed quantitatively via considering the maximum screw shaft and head diameter measurements. Across the 10 pedicle screws placed in the cervical spine of the porcine cadaver, the average maximum screw diameter was measured to be 10.8 ± 0.8 mm, 8.9 ± 1.2 mm and 5.9 ± 0.4 mm for the conventional, sawtooth and double-circle-arc acquisitions, respectively. Screw shaft width comparisons as two sample t-tests between conventional to sawtooth, conventional to double-circle-arc, and sawtooth to double-circle-arc produced p-values of 5e-4, 2e-12, and 4e-7 respectively. The average maximum head diameter was measured to be 13.1 ± 0.3 mm, 13.1 ± 0.4 mm, and 12.9 ± 0.6 mm from the conventional, sawtooth, and double-circle-arc acquisitions respectively. Screw head width comparisons as two sample t-tests between conventional to sawtooth, conventional to double-circle-arc, and sawtooth to double-circle-arc produced p-values of 0.9, 0.5 and 0.6 respectively. The actual maximum screw diameter was 5.0 mm, and the maximum head diameter was 12.0 mm.

**Fig. 4:**
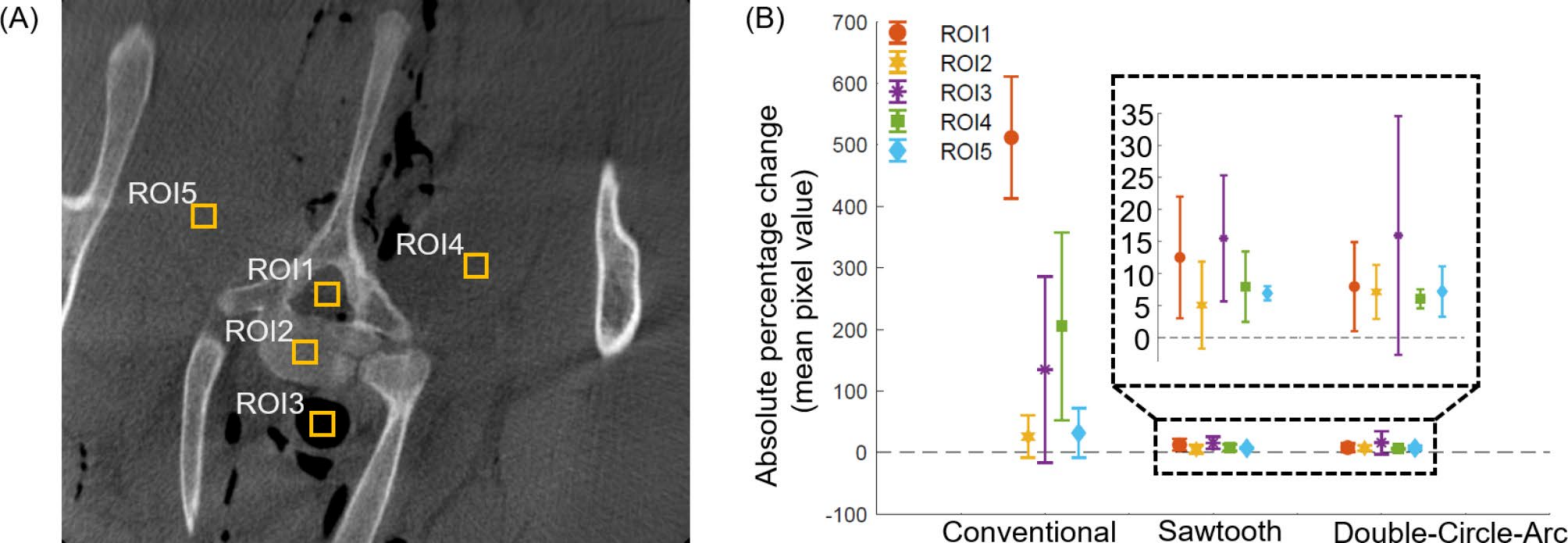
Absolute percentage change in mean pixel values in 5 regions of interest for all vertebral levels containing pedicle screw compared to the ground truth with no metal hardware. (**A**) Ground truth (T1 vertebra, no metal hardware) with the locations of the 5 regions of interest used to calculate the average percentage change in mean pixel values for the reconstructed images following the pedicle screw procedure, labeled ROI1-ROI5. (**B**) Absolute percentage change in mean pixel values in 5 regions-of-interest for all vertebral levels containing pedicle screws (C3-C7) compared to the ground truth (T1, no metal hardware) for the conventional, sawtooth, and double-circle-arc acquisitions.

The average absolute percentage change in the mean pixel values across 5 Regions of Interest (ROIs) for all vertebral levels containing pedicle screws (C3-C7) compared to the ground truth (T1, no metal hardware) from the three acquisition orbits considered is provided in Fig. 4. For the conventional scan, the largest averaged absolute percentage change in mean pixel value was observed in ROI1 located in the spinal canal at 511.7 ± 99.0%, while the smallest averaged absolute percentage change was observed in ROI2 located in the body of the vertebra at 25.8 ± 34.4%. This is confirmed visually with the most significant metal artifacts arising in between the pedicle screws within the spinal canal, as illustrated in Fig. 2. Comparatively for both the sawtooth and double-circle-arc acquisitions, the average absolute percentage changes in mean pixel values across all vertebrae were under 20%. Considering the soft tissue regions, ROI4 and ROI5, the average absolute percentage change in the mean pixel values decreased from the conventional (ROI4 = 205.0 ± 152%, ROI5 = 31.6 ± 40.3%) to the sawtooth (ROI4 = 7.9 ± 5.5%, ROI5 = 6.9 ± 1.1%) and double-circle-arc (ROI4 = 6.0 ± 1.5%, ROI5 = 7.2 ± 3.9%) acquisitions. The average absolute percentage change in mean voxel values in each ROI were compared as two sample t-tests between conventional to sawtooth, conventional to double-circle-arc, and sawtooth to double-circle-arc producing p-values of 0.0004, 0.002 and 0.9 respectively. This is again confirmed visually via the observable streak artifacts in the conventional scans in the surrounding soft tissue around the vertebra.

## Image assessment – double hip arthroplasty

**Fig. 5:**
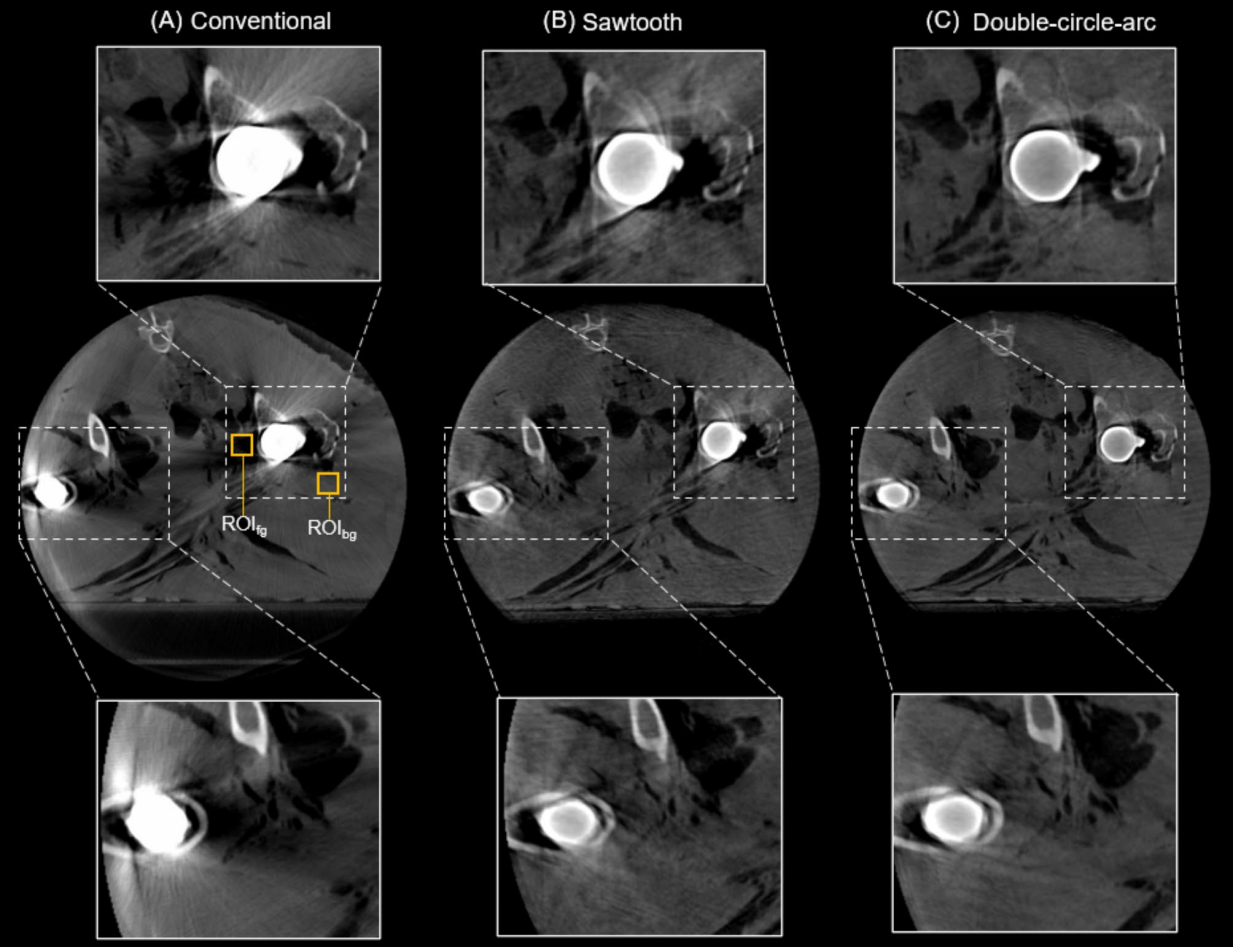
Post-operative reconstructed 3D images (axial view) following double hip arthroplasty in an ovine cadaver. (**A**) Conventional acquisition reconstructed with the in-built metal artifact reduction (MAR) algorithm. The foreground and background Regions of interest, ROI_fg_ and ROI_bg_ respectively, for calculating the contrast-to-noise ratio for all acquisitions are depicted by yellow boxes on the conventional image. (**B**) the sawtooth acquisition and (**C**) the double-circle-arc acquisition. Grey value display: window width (W = 2873) and window level (L = 668).

**Fig. 6:**
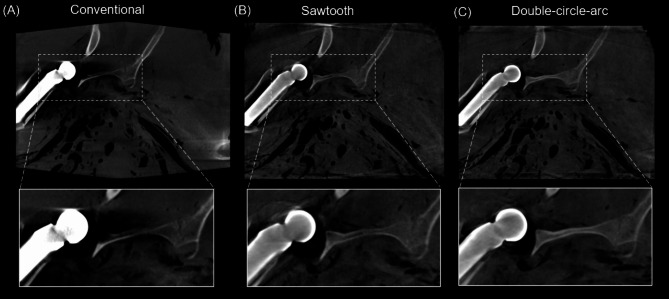
Post-operative reconstructed 3D images (coronal view, left hip) following double hip arthroplasty in an ovine cadaver.** (A)** Conventional acquisition reconstructed with the in-built metal artifact reduction (MAR) algorithm, (**B**) the sawtooth acquisition and (**C**) the double-circle-arc acquisition. Grey value display: window width (W = 2873) and window level (L = 668).

**Fig. 7:**
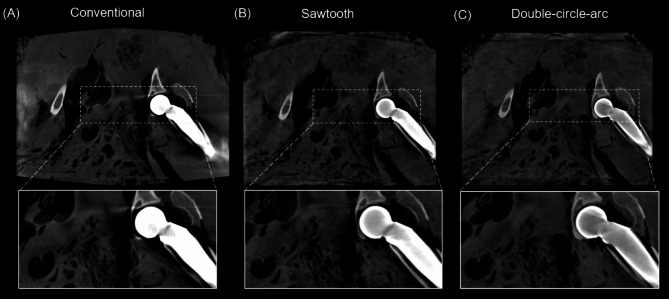
Post-operative reconstructed 3D images (coronal view, right hip) following double hip arthroplasty in an ovine cadaver.** (A)** Conventional acquisition reconstructed with the in-built metal artifact reduction (MAR) algorithm, (**B**) the sawtooth acquisition and (**C**) the double-circle-arc acquisition. Grey value display: window width (W = 2873) and window level (L = 668).

Examples of the 3D reconstructed images from the double hip arthroplasty performed in the ovine cadaver are provided in Figs. 5, 6 and 7 showing the axial, and coronal views of each hip, respectively. Considering the axial view shown in Fig. 5. First, there are significant photon starvation and beam hardening artifacts arising from the hip implants in the conventional scan that are observably reduced with the sawtooth and double-circle-arc acquisitions. Specifically, imaging information of the soft tissue and bone in the proximity of the implants has been recovered with the sawtooth and double-circle-arc acquisitions. In the coronal view of each hip, Figs. 6 and 7, the same improvement in image quality with the sawtooth and double-circle-arc compared to the conventional acquisition can be observed.

The observed reduction in metal artifacts in the sawtooth and double-circle-arc acquisitions was confirmed quantitatively via considering the contrast-to-noise ratio (CNR) near the right implant. The CNR at the right implant, decreased from 17.2 (conventional) to 8.2 (sawtooth) and 7.1 (double-circle-arc), indicating soft tissue close to the implant was recovered with the non-circular acquisitions. We produced contrast weighted voxel values and compared as two sample t-tests between conventional to sawtooth, conventional to double-circle-arc, and sawtooth to double-circle-arc producing p-values of < 2e-16, <2e-16 and 1e-5 respectively.

## Reproducibility of the universal non-circular orbits

**Fig. 8:**
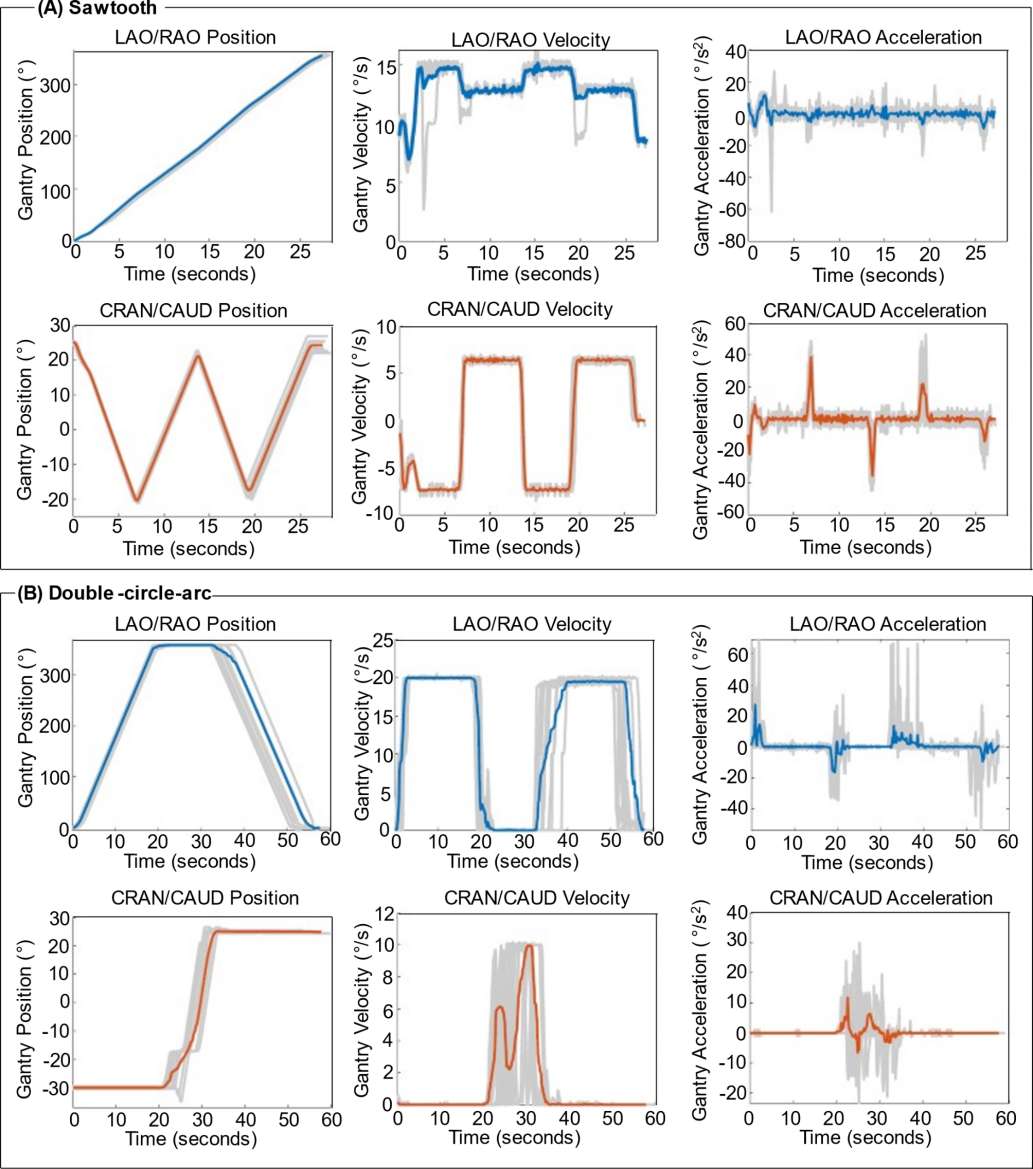
System recorded motion properties of the gantry during the implementation of two universal non-circular imaging orbits. The position, velocity, and acceleration of the C-arm gantry in the left anterior oblique/right anterior oblique (LAO/RAO) and cranial/caudal (CRAN/CAUD) directions from 10 consecutive implementations of (**A**) sawtooth acquisitions and (**B**) double-circle-arc orbits. Colored lines display the average of the 10 implementations (blue and orange), whereas the light grey lines are the individual scans.

The system recorded properties (position, velocity, and acceleration) of the robotic imaging system’s C-arm in the left anterior oblique/right anterior oblique and cranial/caudal directions recorded during 10 consecutive implementations of the sawtooth and double-circle-arc orbits are provided in Fig. 8. Considering first the sawtooth orbit, implemented via additional real-time control hardware. Across the 10 consecutive implementations of the sawtooth orbit, the average scan time was 27.8 ± 0.3 s. The sawtooth orbit was shown to be broadly reproducible, with the left anterior oblique/right anterior oblique positioning displaying high consistency between each acquisition. Observing the cranial/caudal positioning graph in Fig. 8(A) reveals a larger deviation in the final gantry position than for the left anterior oblique/right anterior oblique positioning. The observed deviation is most likely due to system latencies associated with the commands sent to the additional real-time control hardware to reposition the gantry and necessitates the use of online geometric calibration methods to ensure high quality image reconstruction following each acquisition. For the left anterior oblique/right anterior oblique movement, a single command is sent to initiate the clockwise gantry rotation, and another to stop the gantry rotation at the end of the acquisition. Comparatively, four commands are sent to change the cranial/caudal direction of the gantry tilt over the course of the acquisition. Each of the cranial/caudal commands are set to run for a predetermined amount of time to enable the selected frequency (*f* = 2) and angular range (± 25°) for the orbit to be completed in the shortest amount of time possible. However, latencies up to the order of seconds have been observed when sending position commands with the additional real-time control hardware^27,28^, reducing the precision of the orbit.

For the double-circle-arc orbit, across the 10 consecutive implementations of the orbit, the average scan time was 55.9 ± 2.0 s. The double-circle-arc orbit is implemented manually via control of the joysticks on the pilot control module of the robotic imaging system and shown to also be broadly reproducible with the largest deviations occurring in the timing of the second tilted left anterior oblique/right anterior oblique circle. Similarly, to the sawtooth orbit, these variations within subsequent implementations of the orbit necessitate the use of online geometric calibration methods to ensure high quality image reconstruction following each acquisition. The initiation of the second tilted circle was often slightly delayed due to ensuring the correct cranial/caudal tilt (+ 25°) had been reached at the end of the connecting arc. Examining the cranial/caudal position graph in Fig. 8(B), a pause in the C-arm movement is observed in every implementation of the orbit at -17.3°. This pause is due to the knuckle of the C-arm gantry automatically repositioning itself to allow the cranial/caudal movements to be completed and has occurred in every implementation of the double-circle-arc orbit to date.

## Discussion

This study demonstrates the feasibility of implementing universal orbits designed for metal artifact reduction on clinical robotic CBCT systems. Importantly, the universal orbits provided noticeable reduction in the metal artifacts surrounding the two different types of orthopedic hardware considered in this study compared to current conventional imaging. Across both procedures, the double-circle-arc orbit performed the best with the largest quantitative and qualitative reduction in metal artifacts compared to the conventional acquisition. Further reduction of metal artifacts could possibly be achieved by integrating metal artifact reduction reconstruction algorithms into the processing pipeline; however, this study highlights the achievable improvement with acquisition modifications alone.

The universal orbits outlined here are potentially applicable to all fixed-room robotic CBCT systems, regardless of vendor. To implement the sawtooth orbit in the form presented in this study, however, the use of additional real-time control hardware is required. Presently, the additional hardware is only available at one site in the world outside of the vendor. However, with vendor support, a sawtooth orbit for reducing metal artifacts could potentially be made readily available. One example of a non-circular orbit in clinical use is the sine-spin available on the Siemens ARTIS icono. The sine-spin orbit consists of a 200° left anterior oblique/right anterior oblique rotation with a ± 10° cranial/caudal tilt, predominately used for improving image quality during interventional neurology procedures. Comparatively, the double-circle-arc orbit can be implemented via manual control of the system, without the need for additional real-time control hardware. However, additional training of a radiographer or clinician to complete the manual orbit would be potentially required. It is anticipated this process would be a minimal disruption to workflows due to the simple design of the orbit. Vendor support would most likely still be required for widespread clinical use as access to raw/unprocessed 2D projection data is currently required to enable high quality reconstructed images. This is because the exposure parameters of the conventional scan cannot be exactly replicated when implementing the sawtooth and double-circle-arc, or any other non-standard, orbits with the current proof-of-concept implementation strategy. As detailed in previous studies ^27,28,33,34^, presently the only way to facilitate CBCT acquisitions from custom orbits on our system is to acquire 2D X-ray projections with fluoroscopic protocols that have been optimized for 2D imaging and reconstruct 3D volumes using in-house developed reconstruction algorithms. At the beginning of the scan, the exposure parameters for the sawtooth and double-circle-arc orbits are set to align with the conventional acquisition exposure parameters. However, during the scan the exposure parameters fluctuate and deviate from the desired values due to the in-built automatic exposure control for the 2D fluoroscopic protocols. Additionally, the proprietary post-processing of projections that occurs with the conventional 3D acquisition protocols cannot be applied to projections acquired using 2D fluoroscopic protocols, placing a limit on the possible image quality achievable with the universal orbits. Finally, it is not presently possible for our imaging system to perform the 3D image reconstruction from the 2D fluoroscopic X-ray projections acquired during the implementation of the universal non-circular orbits. As such, this necessitates the use of alternative image reconstruction algorithms. Both of the universal non-circular orbits considered in this study were reconstructed with the same algorithm, namely a multiresolution model-based iterative reconstruction with a Penalized Weighted Least-Squares objective and ordered subsets. We still compare our reconstructed images from the universal orbits with the conventional images reconstructed by the imaging system as they remain the current gold standard and understand that what we can currently achieve with our proof-of-concept implementation does not represent the highest image quality that could be achieved with these orbits with increased vendor support.

Another barrier to clinically translating the universal orbits presented in this study is the image reconstruction time. Currently, image reconstruction time is in the order of hours, rendering it not suitable for interventional use. One approach to decreasing the image reconstruction time is utilizing machine learning, with initial studies indicating positive results^29^.

There currently is not a universally used standard metric for quantifying the effect of metal artifacts within CBCT images. Commonly, the effect of metal artifacts is assessed qualitatively through observer ratings and surveys^30,31,32,33,34^. Quantitatively, the effects of metal artifacts are often quantified via some iteration of measuring and comparing implant dimensions, recording variations in HU values and pixel or voxel intensities, and or CNR and signal-to-noise over researcher defined regions of interest in the proximity of the implanted hardware^35,36,37^.

The focus of this study was on two commonly performed orthopedic procedures (pedicle screw fixation and hip arthroplasty), however, due to the universal nature of the orbit designs, the imaging orbits could also be applied to other interventions involving metal hardware. There is also potential for the double-circle-arc orbit to be implemented on mobile C-arm systems, helping to further remove barriers to accessing high quality intraprocedural volumetric imaging. Additionally, we have already begun preliminary investigations into combining motion-compensated reconstruction algorithms with universal orbits to reduce moving metal artifacts during procedures such as cardiac^38^ or tumor ablation^39^ and biopsies^40^ where motion and metal artifacts are present. Future implementations of universal orbits will also include investigating ways to reduce and optimize the projection acquisition to reduce imaging dose to the minimum possible, without compromising on image quality.

## Methods

### Robotic CBCT imaging system and non-circular orbits

A clinical floor mounted robotic CBCT imaging system (Siemens ARTIS pheno, Siemens Healthineers GmbH, Erlangen Germany) was used to implement universal orbits designed to reduce metal artifacts following the completion of two orthopedic procedures on animal cadavers and compared with a circular in-built protocol available on the system known as 5s Dyna CT Body (Siemens Healthineers, GmbH), referred to throughout as the conventional acquisition. The conventional acquisition has a scan time of 5 s, and acquires 397 2D X-ray projections at 90 kV and 0.5 mAs. Two universal orbits were considered, (1) a sawtooth, implemented via additional real-time control hardware provided by Siemens, known as the Siemens Test Automation Control System (TACS)^27,28,41,42^. (2) A double-circle-arc orbit, implemented via manual control of the joysticks on the system’s pilot control module.

The sawtooth orbit was a modification of our previously investigated sinusoidal orbit^22–24^. The change from a sinusoid to a sawtooth was due to the implementation of the orbit with the TACS. The TACS was unable to reliably produce and reproduce a sinusoid orbit due to latencies in the commands to update the gantry position. As such, the sawtooth orbit was implemented by pre-programming the C-arm gantry rotation movements through the TACS using software commands sent via a C# Dynamic Link Library. The sawtooth orbit had a frequency *f* = 2, completing a 360° left anterior oblique/right anterior oblique rotation while reaching a maximum tilt of ± 25° in the cranial/caudal direction. 2D X-ray projections were continuously acquired via the foot pedal during all the acquisition at a set frame rate of 15 frames/second, with 90 kV and 0.5 mAs to match as closely as possible to the conventional acquisition available on the imaging system, resulting in approximately 500 projections. The scan time of the sawtooth orbit was approximately 25 s.

The double-circle-arc orbit was implemented via manual control of the joystick responsible for the C-arm gantry on the system’s pilot control module. The orbit was achieved via the following steps. To initialize the acquisition, the C-arm gantry was manually driven to 0° left anterior oblique/right anterior oblique and tilted to -30° in the cranial/caudal direction. Next, the joystick was manually deflected 100% in the direction controlling the anterior/oblique rotation, driving the C-arm gantry through a 360° clockwise rotation. Note, the specific directions of joystick deflection for each plane of rotation are relative to the orientation of the control module. Following the completion of the first tilted circle, the gantry was manually driven though a cranial/caudal arc from − 30° to + 25° at 0° left anterior oblique/right anterior oblique. Finally, the joystick was manually deflected 100% in the direction controlling the left anterior oblique/right anterior oblique rotation, driving the C-arm gantry through a 360° anti-clockwise rotation. 2D X-ray projections were continuously acquired via the foot pedal during all the acquisition at a set frame rate of 10 frames/second, with 90 kV and 0.5 mAs to match as closely as possible to the conventional acquisition available on the imaging system, resulting in approximately 600 projections. The scan time of the double-circle-arc orbit was approximately 60 s, and is dependent on the operator. The scope to reduce the scan time of the double-circle-arc is currently limited by the maximum gantry rotation velocity when driving the gantry via manual joystick control of 20°/s. For comparison, the current maximum gantry rotation velocity on systems in clinical use is 45°/s.

As access to the additional real-time control hardware used to implement the sawtooth orbit is limited to a single site in the world, outside of the vendor, and the double-circle-arc is implemented via manual control of the imaging system, to demonstrate that the universal orbits can be implemented more than once and to assess the reproducibility of the universal non-circular orbits, the system recorded properties (position, velocity, and acceleration) of the C-arm in the left anterior oblique/right anterior oblique and cranial/caudal directions were recorded during ten consecutive orbit runs. X-ray projections were only acquired on the first of the ten runs producing one set of images for each acquisition type considered (conventional, sawtooth, and double-circle-arc) to be used to complete the image quality assessment.

## Geometric calibration and image reconstruction

As a result of investigating the reproducibility of the sawtooth and double-circle-arc orbits, it was determined that geometric calibration of the orbits required online methods due to the uncertainties in the precise location of each acquired X-ray projection. We tested a 3D-2D registration-based^43^ and a fiducial-based^44^ method on the universal non-circular orbit acquisitions.

The 3D-2D registration-based method used a 3D reconstruction of the conventional circular scan as the registration target. An optimization algorithm iteratively maximizes a gradient-based similarity metric between the measured projections and forward projections from the registration target, thereby finding a system geometry similar to the real geometry. The registration-based method was only applied for the universal non-circular scans of the pedicle screw fixation procedure performed on the porcine cadaver.

The fiducial-based method used spherical fiducials manually placed on the skin of the cadavers. The configuration of the fiducial placement was not measured and hence unknown a priori. The geometric calibration algorithm leverages an assumption: under an accurate system geometry and in 3D world coordinates, lines connecting the locations of the x-ray source throughout a scan and the locations of the projections of a 3D point should intersect. Hence, the algorithm optimizes the estimated system geometry by iteratively estimating the 3D locations of the spherical fiducials and minimizing the distance from these locations to the lines connecting the projection centroids of these fiducials to the x-ray source. The algorithm utilized a fully automatic pipeline to localize fiducials in the projection images and perform iterative geometric calibration. The pipeline ensured robustness in projections where fiducials were impractical to localize due to factors such as overlapping high-density structures (e.g. metal) and field-of-view limitations. This method was only applied for the universal non-circular scans of the double hip arthroplasty procedure performed on the ovine cadaver. 18 steel ball bearings with 4 mm diameter were placed on the skin of the cadaver, and 15 were successfully localized and tracked by the calibration algorithm.

To accommodate the non-circular orbits and minimize truncation artifacts (due to the size of the cadavers and limited field-of-view), we used a multiresolution model-based iterative reconstruction (MR-MBIR) algorithm^45^ with a Penalized Weighted Least-Squares objective and ordered subsets ^46,47^. A low-resolution 3D volume with large voxels and a high-resolution volume with smaller voxels were simultaneously reconstructed. The low-resolution volumes had a relatively small number of voxels (and hence low GPU memory usage) but encompassed a large spatial volume to minimize truncation artifacts. The high-resolution volumes used clinically relevant voxel sizes and were used for image quality evaluation. A quadratic penalty term was applied in the first-order neighborhood of voxels. For both cadaver procedures, the high-resolution 3D volume (used for evaluation) had a dimension of 512 × 512 × 378, and the isotropic voxel size was 0.4737 mm^3^, matching the dimension and voxel size of the reconstructions from the robotic imaging system (Siemens ARTIS pheno, Siemens Healthineers GmbH, Erlangen Germany).

For the pedicle screw fixation in the porcine cadaver, specifically, the low-resolution 3D volume (for reducing truncation artifacts only) had a dimension of 256 × 256 × 189 with 2.8422 mm^3^ isotropic voxels. 500 iterations of multiresolution MR-MBIR were run for both universal non-circular orbits on a computer with an Intel Xeon E5-2620 CPU and an NVDIA RTX TITAN GPU. The reconstruction of the sawtooth acquisition used 5 ordered subsets and took approximately 70 min to complete. The reconstruction of the double-circle-arc acquisition used 8 ordered subsets and took approximately 110 min to complete (due to a higher number of X-ray projections compared to the sawtooth acquisition). Both reconstructions were run simultaneously, so the computation time may be different if they run separately.

For the double hip arthroplasty in the ovine cadaver, specifically, the low resolution 3D volume dimension was 128 × 128 × 92 with 3.7896 mm^3^ isotropic voxels. 200 iterations of MR-MBIR were run for both non-circular orbits on a computer with an AMD Ryzen 9 5950x CPU and an NVDIA RTX 3090 Ti GPU. The reconstruction of the sawtooth acquisition used 5 ordered subsets and took approximately 7 min to complete. The reconstruction of the double-circle-arc acquisition used 5 ordered subsets and took approximately 13 min to complete (due to a higher number of X-ray projections compared to the sawtooth acquisition).

The conventional acquisitions were reconstructed with the in-built metal artifact reduction algorithms provided on the imaging system.

## Pedicle screw fixation

A 5-level pedicle screw fixation in the cervical spine (C3-C7) of a porcine cadaver was performed by an experienced orthopedic surgeon. The pedicle screws were provided by Evolution Surgical (Sydney, Australia) and had maximum shaft diameters of 5.5 mm, length of 30 mm, and head diameter of 12.0 mm. Custom designed 3D-printed pedicle screw drill guides were used during the procedure. Details of generating the pedicle screw drill guides used in this study from image acquisition and processing, vertebrae segmentation, pedicle screw trajectory planning, through to 3D-printing are provided elsewhere ^48^.

The porcine cadaver was placed in a prone position and a midline posterior incision and approach used. For the C3-C7 vertebrae, a subperiosteal dissection was performed. Next, the area surrounding the vertebrae where the guides were to be positioned were cleaned of soft tissues, with care taken to not disturb the facet joint capsule and bone. Stability of each individual drill guide was confirmed with gently downward digit pressure. A 4.2 mm drill bit was used to create a pilot hole for the pedicle screws. A ball-tipped pedicle probe was used to confirm no palpable breach. The templated pedicle screw was then inserted.

To quantitatively assess the reconstructed image quality from the three acquisition methods considered surrounding the pedicle screws, screw dimension measurements and absolute percentage change in mean pixel values across 5 regions-of-interest (ROIs) were used. For the screw dimension measurements, the maximum diameter of the shaft and head of each screw was recorded and compared between acquisitions and the diameters provided by the manufacture^35^. For the absolute percentage change in mean pixel values 5 ROIs were selected, where each ROI was 10 × 10 mm^2^ square in the plane of the pedicle screws, Fig. 4. The ROIs were selected to allow observation of the absolute percentage change in the mean pixel value across various anatomical structures within and surrounding the vertebra. Specifically, ROI1 was selected to reside within the spinal canal, ROI2 was selected to reside within the vertebral body, ROI3 was selected to reside within the esophagus, and ROI4 and ROI5 were selected to reside within the neighboring soft tissue. As the reconstructed 3D volumes had matching dimensions and isotropic voxel sizes, the pixel and slice locations of each ROI was consistent across all volumes considered. The T1 vertebra was used as the ground truth as no pedicle screws were placed at this vertebral level. The absolute percentage change in mean pixel values of the 5 ROIs was then calculated for each vertebral level and averaged to give a single value per acquisition considered (conventional, sawtooth, and double-circle-arc).

### Hip arthroplasty

A double hip arthroplasty of an ovine cadaver was performed by an experienced orthopedic surgeon. Custom designed 3D-printed titanium and stainless-steel hip prostheses were provided by OSSIS Corporation (Christchurch, New Zealand). The cadaver was placed in the lateral position for each hip placement. A lateral incision and classical posterior approach to the hip was utilized. The hip joint was dislocated posteriorly, and the femoral neck was osteotomized. The femoral canal was perforated and sequentially broached to the appropriately sized implant. A custom, uncemented implant was inserted and the hip was reduced. Identical procedures were performed bilaterally. The fascia and skin were closed in a standard fashion.

To quantitively assess the reconstructed image quality from the three acquisition methods considered surrounding the hip prothesis, the metric of contrast-to-noise (CNR) in regions in close proximity to one of the prothesis was used ^36,37^. Here, a lower CNR value would indicate better image quality due to more soft tissue being resolved close to the implant in the region most noticeably impacted by the metal artifacts. Absolute percentage change in mean pixel value calculations, as used for the pedicle screw fixation procedure, were not applicable to the hip procedure as there was no similar anatomical region within the volume to use as a ground truth. That is, the T1 vertebra was able to be used as a ground truth reference as no pedicle screws were placed at that vertebra level, however, both hips had a prothesis placed within them.

The CNR was calculated using:

1 $$\:CNR=\frac{|{\mu\:}_{ROIfg}-{\mu\:}_{ROIbg}|}{\sigma\:}$$

Where µ_ROIfg_ and µ_ROIbg_ were the mean pixel values of the foreground (ROI_fg_) and background (ROI_bg_) 10 × 10 mm^2^ regions of interest respectively, as shown in Fig. 5, and σ was the standard deviation within the background region of interest. Note that CNR is dimensionless but can be understood as the number of standard deviations separating the foreground and background mean values. ROI_fg_ was selected to reside adjacent to the right prothesis, in the region most heavily impacted by the metal artifacts arising from the prothesis, while ROI_bg_ was selected to reside in a region of soft tissue in proximity to the prothesis but relatively unimpacted from the metal artifacts. The CNR was only calculated near one prothesis to limit the influence of any bone structures on the calculation.

### Animal preparation

The porcine and ovine cadavers used in this study were directly obtained from the Laboratory Animal Services at the University of Sydney, Australia, in accordance with the University’s Animal Ethics Procedures following the conclusion of two previous and separate studies. The previous studies required and included ethics to sacrifice the animals, which were obtained through the University’s Animal Ethics Committee. The animals were not sacrificed for the purpose of completing this study.

## Data Availability

The datasets used and/or analysed during the current study available from the corresponding author on reasonable request.
